# Where is mineral ballast important for surface export of particulate organic carbon in the ocean?

**DOI:** 10.1002/2014GL061678

**Published:** 2014-12-03

**Authors:** Frédéric A C Le Moigne, Katsiaryna Pabortsava, Charlotte L J Marcinko, Patrick Martin, Richard J Sanders

**Affiliations:** 1 Ocean Biogeochemistry and Ecosystems, National Oceanography CentreSouthampton, UK; 2 School of Ocean and Earth Science, University of SouthamptonSouthampton, UK; 3 Earth Observatory of Singapore, Nanyang Technological UniversitySingapore

## Abstract

Correlations between particulate organic carbon (POC) and mineral fluxes in the deep ocean have inspired the inclusion of “ballast effect” parameterizations in carbon cycle models. A recent study demonstrated regional variability in the effect of ballast minerals on the flux of POC in the deep ocean. We have undertaken a similar analysis of shallow export data from the Arctic, Atlantic, and Southern Oceans. Mineral ballasting is of greatest importance in the high-latitude North Atlantic, where 60% of the POC flux is associated with ballast minerals. This fraction drops to around 40% in the Southern Ocean. The remainder of the export flux is not associated with minerals, and this unballasted fraction thus often dominates the export flux. The proportion of mineral-associated POC flux often scales with regional variation in export efficiency (the proportion of primary production that is exported). However, local discrepancies suggest that regional differences in ecology also impact the magnitude of surface export. We propose that POC export will not respond equally across all high-latitude regions to possible future changes in ballast availability.

## 1 Introduction

The biological carbon pump (BCP) is an important component of the global carbon cycle, exporting 5 to 10 Gt C yr^−1^ from the surface ocean to the ocean's interior [*Henson et al.*, 2011]. In the deep ocean, strong correlations are observed between fluxes of particulate organic carbon and minerals [*Klaas and Archer*, 2002]. Therefore, it has been suggested that fluxes of minerals drive organic carbon flux, either by increasing particle density and thus sinking speed or possibly by physically protecting a proportion of the particulate organic carbon (POC) flux that leaves the surface from degradation [*Armstrong et al.*, 2002; *François et al.*, 2002; *Klaas and Archer*, 2002]. Several ocean models now use a mineral protection model to describe the flux of material to depth [*Gehlen et al.*, 2006; *Yool et al.*, 2013].

An obvious question to ask is whether similar patterns are seen in the surface ocean. Our previous analysis, based on limited data sets derived from ^234^Th disequilibria, revealed linear relationships between shallow export of organic carbon and biominerals and further showed that exported material appeared to be biomineral-rich relative to the average composition of surface particulate matter [*Sanders et al.*, 2010]. This suggests that a ballast effect is probably important in the surface export of POC. A much larger data set subsequently revealed considerable scatter in the relationship between mineral and POC export, suggesting that regional differences in ecology strongly impact particle flux [*Le Moigne et al.*, 2012]. In particular, we suggested that the unballasted pool can be large in diatom-dominated regions like the Southern Ocean (SO), which might explain the low transfer efficiencies (ratio of deep export over surface export, *T*_eff_) reported by some studies in such regions [*Le Moigne et al.*, 2012].

Further analysis of the global data set of deep particle flux by *Wilson et al.* [2012] has shown that the use of geographically weighted regression analysis (GWR), which allows model parameters to vary spatially, is more appropriate than the single multiple linear regression used by *Klaas and Archer* [2002] and *Le Moigne et al.* [2012] and others. GWR reveals spatial patterns in mineral carrying coefficients (Ccs, the statistical coefficients of a multiple regression model of POC flux versus biomineral flux; see section 2), which are likely driven by spatial variability in pelagic ecology [*Wilson et al.*, 2012]. *Wilson et al.* [2012] hypothesized that the variations observed in deep Ccs match the global pattern in *T*_eff_; namely, that *T*_eff_ is high in calcite-dominated regions and low in opal-dominated regions. While this is the global *T*_eff_ pattern reported by some studies [*François et al.*, 2002; *Henson et al.*, 2012; *Lam et al.*, 2011], shipboard process studies have revealed that diatom blooms can lead to large export events characterized by high *T*_eff_ [*Buesseler and Boyd*, 2009; *Martin et al.*, 2011; *Rynearson et al.*, 2013; *Smetacek et al.*, 2012]. This basic disagreement about the effect of opal ballast underscores how limited our understanding of mineral ballast in the oceans is.

However, it is still unclear how the impact of ballasting on shallow POC export varies regionally and how it is linked to export efficiency (defined as shallow export over primary productivity, PE_eff_). Here we examine the variability in surface mineral ballast Ccs using ^234^Th-derived estimates of POC and mineral flux in the Arctic, Atlantic, and Southern Oceans (95 data points). We discuss the implications of our results for the role of minerals in the export of POC from the surface ocean.

## 2 Material and Methods

### 2.1 Flux Data

We updated our POC and mineral export database derived from ^234^Th measurements published in *Le Moigne et al.* [2012] with data from six additional cruises (JC068, D357, D361, D369, JR271, and JR274). This extended data set includes observations from the Arctic ocean, the high-latitude North Atlantic (HLNA), the equatorial Atlantic, and the SO (Figure S1 in the supporting information). Locations, sampling dates, and references are given in Table S1 (supporting information). We used the ^234^Th “small-volume” technique with inductively coupled plasma–mass spectrometry measurement of ^234^Th extraction efficiency [*Pike et al.*, 2005]. Vertical profiles of ^234^Th activity were integrated to 100 m and converted to estimates of downward ^234^Th flux using a one-dimensional steady state model [*Buesseler et al.*, 1992].

These fluxes were then converted to estimates of downward particle flux using the POC:^234^Th or mineral:^234^Th ratio of large (>53 µm) particles collected on Nitex screen using an in situ stand-alone pumping system deployed at a single depth beneath the mixed layer (from 20 to 70 m). Particles were then rinsed off the screen using thorium-free seawater, and the particle suspension split into equal subsamples. Each split was then analyzed for one of the following: ^234^Th, POC, BSi, and particulate inorganic carbon (PIC) as in *Le Moigne et al.* [2013b].

Using the ^234^Th approach has the great advantage of providing a large data set of surface export fluxes, albeit with the caveat that some of the seasonal variability of export flux may be missed. While this is unlikely to be a major issue in low-latitude areas where the seasonal cycle in export is weak [*Waniek et al.*, 2005], at high latitudes, where seasonal variability is stronger, our Th-derived export fluxes may have missed the episodes of large export often observed in such environments [*Waniek et al.*, 2005].

The aluminum concentration in large particles (PAl) was not measured; therefore, literature values were taken from *Lambert et al.* [1984] and *Kuss and Kremling* [1999] to estimate downward fluxes of Al following *Le Moigne et al.* [2012] and *Honda and Watanabe* [2010]. For the JR274 cruise in the Scotia Sea, we used the average PAl (>53 µm) found in the plume of the Crozet Islands [*Planquette et al.*, 2009] (PAl = 1.27 nmol l^−1^) for stations in the plumes of chlorophyll *a* associated with South Georgia and the South Sandwich Islands and the average value from nonplume stations around Crozet [*Planquette et al.*, 2009] (PAl = 0.23 nmol l^−1^) for our nonplume stations. Table S1 summarizes the values of PAl used for each station. Lithogenic fluxes were estimated as (100/8*Al) [*Honda and Watanabe*, 2010] assuming that most PAl is of lithogenic origin, although it can be present in diatom frustules [*Gehlen et al.*, 2002]. All the flux data are listed in Table S1. Using literature values for PAl concentration to estimate the flux of lithogenics may introduce inaccuracies and represents a limitation of our method. However, lithogenic flux is a minor component of the flux and is generally much lower than the biomineral fluxes [*Salter et al.*, 2010]. The errors associated with our estimates of lithogenic flux therefore have only a limited impact on our conclusions.

### 2.2 Regression Model and Geographically Weighted Regression

We examined the relationship between mineral and POC fluxes using two different techniques. Initially, we used multiple linear regression analysis (MLRA) as in *Honda and Watanabe* [2010] and *Le Moigne et al.* [2012]. MLRA divides total POC flux (POC_total_ flux) into four portions, three of which are considered to be associated with the mineral phases BSi (POC_BSi_), calcite (POC_pic_), and lithogenic material (POC_lith_). The fourth portion is the residual POC flux, which is considered the non-associated fraction (POC_res_). MLRA takes the following form:


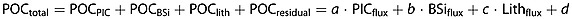
where *a*, *b*, and *c* are defined as Ccs [*Klaas and Archer*, 2002], while *d* is the nonassociated portion of POC [*Armstrong et al.*, 2002; *Honda and Watanabe*, 2010; *Le Moigne et al.*, 2012].

We then used the geographically weighted regression (GWR) technique. The application of GWR to ocean particle flux data was carefully described and tested by *Wilson et al.* [2012]. We followed a similar approach to estimate geographically weighted carrying coefficients for each of the ballast components and the fraction of POC not associated with any mineral. In essence, the GWR analysis calculates “local carrying coefficients” at each sampling location for the ballast minerals and the residual, nonassociated POC [*Wilson et al.*, 2012].

We used the GWR 4.0 software [*Nakaya et al.*, 2009] (freely available at http://www.st-andrews.ac.uk/geoinformatics/gwr/gwr-software/). We used an adaptive Gaussian Kernel, which groups the data by the nearest neighbor approach. The Akaike Information Criterion, corrected for small sample bias (AICc) [*Akaike*, 1974], was used to define the optimal bandwidth following *Wilson et al.* [2012]. We also carried out a test of geographical variability (TGV) to test the spatial variability of the local Ccs provided by the GWR.

## 3 Results and Discussion

### 3.1 POC and Mineral Export Fluxes

**Figure 1:**
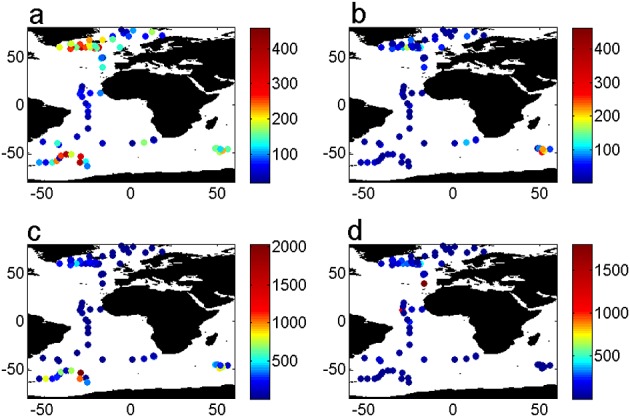
(a) POC, (b) PIC, (c) BSi, and (d) lithogenic material export (in mg m^−2^ d^−1^) as a function of latitude and longitude (°).

In the following section, we briefly describe the magnitude of surface POC and mineral export fluxes (Figure 1). The highest levels of POC export are found in subpolar regions, with values in the Arctic being lower (Figure 1a). Rates of POC export are higher in the North Atlantic oligotrophic gyre (between 25°N and 0°N) than in the South Atlantic oligotrophic gyre (between 25°S and 0°S) (Figure 1a).

PIC export (Figure 1b) shows similar trends, with the Southern Hemisphere subpolar fluxes being lower than the Northern Hemisphere ones. The large PIC export fluxes in the SO are all located around the Crozet islands. The export of BSi (Figure 1c) shows similar patterns with subpolar fluxes in the Northern Hemisphere being larger than those in the Southern Hemisphere. The export flux of BSi in the Atlantic is mostly restricted to subpolar latitudes, as our data set does not include any diatom-dominated upwelling environments. Lithogenic export fluxes (Figure 1d) are very high at some of the northern subtropical gyre stations, moderate in parts of the subpolar North Atlantic, and low elsewhere. At midlatitudes, POC export still occurs while biomineral export is low. Mineral and organic carbon export fluxes are relatively low in the Arctic Ocean, reflecting the oligotrophic to mesotrophic character of the cold northern seas.

### 3.2 Results From MLRA and GWR

The MLRA yielded an *R*^2^ of 0.22 and an AICc of 1139 (Table 1). The GWR increased the *R*^2^ to 0.52 and reduced the AICc to 1098 (Table 1), indicating that the GWR described significantly more variability than the MLRA. Moreover, a comparison of correlograms (used to indicate the presence of autocorrelation at different spatial extents) for the MLRA and GWR model residuals indicated that the GWR model removed all significant spatial autocorrelation. The GWR model therefore provides a more unbiased estimate of the error associated with the Ccs when compared to the MLRA model estimates, which are significantly affected by spatial autocorrelation at multiple spatial scales (see Figure S2 in the supporting information). An analysis of variance analysis showed that the GWR model is a significant improvement on the global MLRA (F(91,84) = 9.4, *p* < < 0.001). Formally, we reject the null hypothesis that there is no difference between the GWR and MLRA models. Finally, the test of geographical variability (TGV, Table 1) revealed significant spatial variation in the local Ccs for PIC and BSi (Diff of Criterion < −2, indicating that variation in Ccs was significant), while there was only weak evidence of spatial variation in the Cc for lithogenic material. The GWR analysis yielded an optimal bandwidth [*Nakaya et al.*, 2009] equal to 30 (this number indicates the sample size over which the GWR fits the regression locally) and thus includes interbasin influences [*Wilson et al.*, 2012]. This effect was limited, however, as most of our data are concentrated in the Atlantic (Figure S1). We note that some calculated Ccs include the influence of data from quite distant locations. This is particularly true for Ccs obtained in the eastern equatorial south Atlantic, which were influenced by export fluxes measured in the Southern Ocean. This highlights the need for more export measurements from this specific area (Figure 1).

**Table 1:** Carrying Coefficients and Statistical Significance

	Carrying Coefficients^a^				Statistical Significance			
	PIC	Bsi	Lithogenic	Intercept^b^				
	*a*	*b*	*c*	*d*	AICc^c^	*R*^2^	*n*	*p* Value
MLRA	0.25	0.15	0.08	105.33	1139	0.224	95	<0.001
GWR^d^,^e^	0.57 (−6.3)	0.26 (−2.2)	0.10 (−0.6)	89.00 (3.6)	1098	0.52	95	<0.001
Associated range for local CCs^f^	1.22	0.27	0.27	14.17	-	-	-	-

a As described in section 2.

b In mg m^−2^ d^−1^.

c Following *Akaike* [1974].

d Calculated using the “optimal bandwidth” [*Nakaya et al.*, 2009].

e Values in brackets are the “Diff of Criterion” resulting from the test of geographical variability (TGV; see section 2). This allows an assessment of whether each regression coefficient is varying over space. If the Diff of Criterion is a positive value, this suggests no spatial variability in the local term. If the difference of AICc is less than two, there is no essential difference. If the Diff of Criterion is between −2 and +2, this suggests only “weak support” of the model comparison.

f Range between the highest and lowest values observed from the local Ccs.

Henceforth, we discuss only the results of the GWR analysis. These results significantly increase the spatial coverage of our previous work [*Le Moigne et al.*, 2012] and demonstrate that GWR is an appropriate tool to analyze surface flux data.

**Figure 2:**
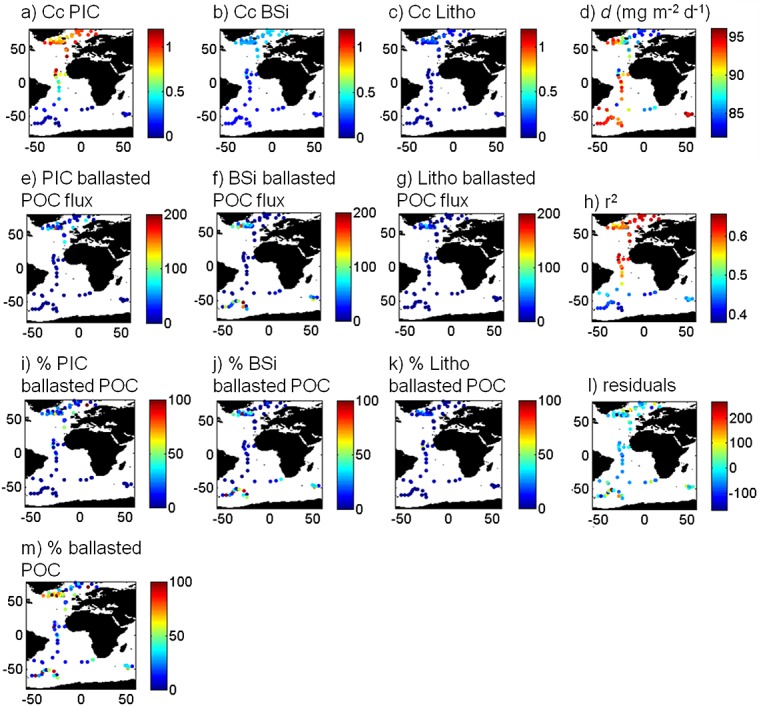
Geographically weighted carrying coefficients for (a) PIC, (b) BSi, and (c) lithogenic material. (d) Intercept (*d* or nonmineral-associated fraction; see section 2), (e) PIC-associated POC export (mg m^−2^ d^−1^), (f) BSi-associated POC export (mg m^−2^ d^−1^), (g) lithogenic-associated POC export (mg m^−2^ d^−1^), (h) *R*^2^, (i), percentage of POC export ballasted by PIC, (j) percentage of POC export ballasted by BSi, (k) percentage of POC export ballasted by lithogenic material, (l) model residuals, and (m) percentage of ballasted POC export.

The GWR-derived PIC and BSi Ccs display some clear spatial trends, while lithogenic Ccs were less variable (Figure 2), as also indicated by the TGV (Table 1). However, all the Ccs differ substantially from the mean global values (Table 1), showing that a single global parameterization of a ballast mineral model is not an accurate description of shallow POC export.

GWR Ccs for PIC are high in the North Atlantic subtropical gyre, the HLNA, and the Nordic Seas (Figure 2a), with lower values seen in the South Atlantic subtropical gyre and the SO. The largest variability in Ccs is observed near the equator (Figure 2a). Ccs for BSi follow a similar latitudinal pattern to those for PIC but are less variable (Figure 2b). Finally, Ccs for lithogenic material vary very little (Figure 2c) but also tend to increase northward. The intercept (*d*) is high in the SO as previously observed [*Le Moigne et al.*, 2012] but is otherwise relatively constant across the data set, between 80 and 90 mg m^−2^ d^−1^, with the lowest values seen in the Arctic and the North Atlantic subtropical gyre (Figure 2d and Table 1).

The GWR model fit the data better in the HLNA than in the SO and the Southern Atlantic (Figure 2h). This could be an artefact of having more data in the HLNA and Arctic than in the SO and Southern Atlantic (24 data points south of 45°S and 49 data points north of 45°N), as a higher density of data allows for a better model fit [*Nakaya et al.*, 2009]. *Wilson et al.* [2012, Figure 6] also observed a high correlation coefficient in regions with high sample density, such as the Arabian Sea, equatorial Pacific, and the sub-Arctic Pacific.

To ease the interpretation of the different ballast Ccs we obtained (Figures 2a–2d), we calculated the flux of POC associated with each mineral and the proportion of total POC flux associated with each mineral. The PIC-associated POC flux and the proportion of total POC flux associated with PIC are both highest in the HLNA (Figures 2e and 2i). The POC flux associated with BSi is the largest in the SO, although stations in the HLNA also have elevated BSi-associated POC fluxes (Figure 2f). The lithogenic-associated POC flux is low relative to the flux associated with the biominerals (Figure 2g). On average and globally, the proportions of total POC flux associated with PIC and lithogenics (14 and 6%, respectively) are smaller than the proportion of total POC flux associated with BSi (21%).

The proportion of POC flux associated with mineral ballast is highest in the HLNA and the SO (Figure 2m). With the exception of one station in the Barents Sea, the proportion of POC flux carried by biominerals is generally low in the Arctic Ocean. The high fraction of mineral-associated POC export at the Barents Sea station is likely due to a coccolithophore bloom often observed in this region [*Smyth et al.*, 2004]. The proportion of total POC flux carried by each mineral (Figures 2i–2k) shows similar trend as described above. The fraction of export not associated with biominerals shows a wide range, often comprising the majority total POC export (62% on average), for example, in the transition regions between subpolar and subtropical environments (Figure 2m).

Overall, our analysis suggests that the ballasted fraction varies spatially and is highest in the HLNA (60% on average, mostly PIC and BSi) and SO (38% on average, mostly BSi) (Figures 2i, 2j, and 2m). Most of the export at low latitudes and in the Arctic is not associated with minerals.

### 3.3 Does Mineral Ballasting Help to Explain Global Patterns in POC Export Efficiency?

To fully understand the interaction between mineral and POC fluxes, one needs to assess the variation of mineral-associated POC fluxes not only at depth [*Wilson et al.*, 2012] but also in surface waters. This is because (1) the mineral content of particles exported from the surface does not necessarily reflect the average mineral content of suspended particles in surface waters [*Le Moigne et al.*, 2012] and (2) the dissolution/remineralization rates of the various POC and mineral phases do not necessarily covary in the water column [*Milliman et al.*, 1999; *Ragueneau et al.*, 2006], implying that the mineral Ccs in the surface ocean may differ from those in the deep ocean [*Wilson et al.*, 2012].

It has been suggested that minerals influence the transfer efficiency, *T*_eff_, of flux through the mesopelagic [*François et al.*, 2002]. To investigate whether there might also be an association between the proportion of POC export that is mineral-associated and the export efficiency, PE_eff_, we now compare our mineral-associated POC export data to the global pattern of PE_eff_ reported by *Siegel et al.* [2014].

*Siegel et al.* [2014] report the highest PE_eff_ in their data set from the HLNA (0.1–0.25), where our analysis shows the highest proportion PIC- and BSi-associated POC export (Figures 2i–2k). This suggests that in the HLNA, BSi and PIC do play a role in promoting POC export, as previously suggested by *Sanders et al.* [2010] and *Buesseler and Boyd* [2009].

Conversely, PE_eff_ is generally lower in the SO (0.01–0.15), where a lower proportion of the total POC export is mineral associated than in the HLNA (Figure 2m). Hotspots of high PE_eff_ in the SO occur mainly in the intense blooms stimulated by iron release from islands such as Kerguelen, Crozet, and South Georgia [*Siegel et al.*, 2014]. Export rates in these regions are enhanced relative to iron-limited areas of the SO (Figure 1a); however, the proportion of ballast-associated POC (mainly BSi-associated) does not vary significantly between the fertilized and the iron-limited regions (39 and 42%, respectively, Figure 2m).

In the subtropical gyres, where the spatial variability of mineral-associated POC export is reduced and the proportion of the POC flux associated with minerals is low (Figures 2e–2g and 2m), PE_eff_ is moderate (0.05–0.15) [*Siegel et al.*, 2014].

The proportion of POC flux carried by mineral does not so clearly correlate with the PE_eff_. While we can only compare our export data to the climatology of PE_eff_ reported by *Siegel et al.* [2014] (and not directly with PE_eff_ at the time of sampling), the comparison suggests the following:

1. There are fundamental differences in the ways minerals promote POC export in different oceanic provinces. We know that mineral ballast can have different ballasting properties (particle nucleation and/or added density) depending on mineral type. For example, calcite-ballasted particles generally sink faster than opal-ballasted ones [*Iversen and Ploug*, 2010]. However, calcite originating from coccolithophores is more likely to ballast POC efficiently than calcite originating from foraminifera [*Schmidt et al.*, 2014]. This implies that there is no globally uniform relationship between export of one type of mineral and POC, contrary to earlier suggestions by *Klaas and Archer* [2002] and *Sanders et al.* [2010].
2. Factors other than the magnitude of mineral-associated POC flux are also likely to influence PE_eff_. For instance, ecosystem functions such as the packaging of slowly sinking phytoplankton cells into large, fast-sinking fecal pellets may increase PE_eff_ regardless of the proportion of POC associated with minerals in the material grazed.

We suggest that these processes are potentially more important in the SO and the subtropical gyres than in the HLNA. This is consistent with observations of an inverse relationship between primary production and PE_eff_ in the SO, potentially due to grazing intensity [*Maiti et al.*, 2013], and the presence of large amounts of fecal pellets found in deep sediment traps located in the centers of the subtropical gyres [*Pabortsava*, 2014].

### 3.4 Implications

Our analysis reveals two important messages:

1. Globally, POC not associated with minerals often dominates total POC export out of the surface ocean (Figure 2k). While it is quite clear that this flux gets remineralized with increasing depth in the mesopelagic zone [*Armstrong et al.*, 2002; *Honda and Watanabe*, 2010], what drives the size of this unassociated flux in surface waters is unclear. As it is often a large proportion of the POC flux, it is important to understand the formation mechanism of this pool and how it may vary with future changes in the ocean. This pool may indeed play a role in setting POC remineralization depth, which has the potential to exert strong control over atmospheric CO_2_ levels [*Kwon et al.*, 2009].
2. Surface mineral-associated POC fluxes are highly variable (Figures 2i–2k), but some of the variability correlates to a certain extent with the patterns of PE_eff_ presented by *Siegel et al.* [2014], suggesting that PIC and BSi can promote efficient export of POC. Our results suggest that this is most pronounced in the HLNA. Conversely, our results suggest that mineral ballast association may be of limited importance in explaining the variation of PE_eff_ in the SO and the subtropical gyres. Ecosystem processes like grazing intensity may be more important in these regions.

Comparing mineral-associated POC export with the global pattern of PE_eff_, *Siegel et al.* [2014] has provided valuable information to specifically diagnose the local role of minerals. The comparison can also yield insights into regional patterns in *T*_eff_.

In the HLNA, estimates of *T*_eff_ derived from sea surface temperature are low [*Henson et al.*, 2012]. However, the large mineral-associated surface flux of POC we observe in the HLNA is likely to sink fast and to be protected from remineralization in the mesopelagic [*Engel et al.*, 2009a; *Engel et al.*, 2009b; *Le Moigne et al.*, 2013a]. Hence, it is possible that *T*_eff_ in the HLNA is higher than proposed by *Henson et al.* [2012], as was indeed shown during process studies upon collapse of spring diatom blooms in the HLNA [*Buesseler and Boyd*, 2009; *Martin et al.*, 2011; *Rynearson et al.*, 2013]. In the SO, *T*_eff_ is also estimated to be low [*Henson et al.*, 2012], and since the proportion of mineral-associated POC flux is lower than in the HLNA, we propose that the POC is less protected and thus potentially more easily degraded by consumers. In this case, our results are in keeping with recent literature arguing that ecosystem structure is more important than ballast mineral in driving the *T*_eff_ in high-latitude diatom-dominated regions [*Assmy et al.*, 2013; *Henson et al.*, 2012; *Lam et al.*, 2011; *Wilson et al.*, 2012], although in the SO, too, it has been shown that the collapse of a diatom bloom can lead to a large export event characterized by high *T*_eff_ [*Smetacek et al.*, 2012].

Our results suggest that viewing the BCP as a two end-member system with high PE_eff_/low *T*_eff_ occurring at high latitudes and low PE_eff_/high *T*_eff_ occurring at low latitudes [*Henson et al.*, 2012] may miss some of the complexity inherent within the system. Rather, the processes driving PE_eff_ and *T*_eff_ are likely to be driven in turn by factors beyond just sea surface temperature. Thus, not all high-latitude regions are identical, and they cannot be described by the same “ballast” parameterization in ocean carbon models. For instance, POC export in the HLNA and the SO are unlikely to respond in the same way to any reduction in ballast mineral availability, as might be caused by ocean acidification, increased sea temperature, or deepening of the wind mixed layer [*Sarmiento et al.*, 2004; *Zondervan*, 2007]. We propose that the BCP in the HLNA may be more susceptible to reductions in ballast mineral availability than the BCP in other high-latitude regions in the world ocean. As the HLNA is a major contributor to global POC export [*Falkowski et al.*, 1998], any reduction in the export of POC there could have a significant impact on the level of atmospheric CO_2_.

## Key Points

- Nonballast-associated POC often dominates the POC flux in the surface ocean
- Highest fraction of mineral-associated POC export in the North Atlantic
- High-latitude areas will not respond equally to changes in ballast availability

## Supporting Information

Readme
