# Supplementary material for: Where is mineral ballast important for surface export of particulate organic carbon in the ocean?
Source: Geophys Res Lett. 2014 Dec 3;41(23):8460–8. doi: 10.1002/2014GL061678 (PMC4459180; doi:10.1002/2014GL061678)
Supplement: Supplementary file 1 — Readme [file grl0041-8460-sd1.docx]

Where is mineral ballast important for surface export of particulate organic carbon in the ocean?

Frédéric A.C. Le Moigne^1*^, Katsiaryna Pabortsava^2^, Charlotte L.J. Marcinko^1^, Patrick Martin^2,3^, Richard J. Sanders^1^.

^1^Ocean biogeochemistry and Ecosystems, National Oceanography Centre, Southampton, SO14 3ZH, UK; ^2^School of Ocean and Earth Science, University of Southampton, Southampton SO14 3ZH, UK. ^3^Earth Observatory of Singapore, Nanyang Technological University, 50 Nanyang Avenue, Singapore 639798.

**:* Corresponding author, Frédéric A.C. Le Moigne, [f.lemoigne@noc.ac.uk](mailto:f.lemoigne@noc.ac.uk); National Oceanography Centre, European Way, SO143ZH, Southampton, U.K.

Geophysical Research Letters, 2014

Introduction

Additional table and figures referred in the main manuscript are described here.

All of these elements are included in a single file entitled “README Le Moigne et al 2014”. Table S1 presents the stations positions, the fluxes and the associated reference. Figure S1 maps the station locations. Figure S2 shows correlograms comparing the spatial autocorrelation across station distance.

Supplementary Table S1: Compilation of Th derived POC and mineral export fluxes data used in the regression analysis.

1.1 Column “Longhurst, 1991, provinces” give the corresponding “Longhurst” province of the station

1.2 Column “Latitude” gives the latitudes of the stations

1.3 Column “Longitude” gives the longitudes of the stations

1.4 Column “POC export” gives the export flux of POC in mg m^-2^ d^-1^

1.5 Column “PIC export” gives the export flux of PIC in mg m^-2^ d^-1^

1.6 Column “BSi export” gives the export flux of BSi in mg m^-2^ d^-1^

1.7 Column “Litho export” gives the export flux of lithogenic material in mg m^-2^ d^-1^

1.8 Column “Surface temperature” gives the temperature of the surface water at each location

1.9 Column “PAl” gives the concentration of Al in large particles used to estimate the flux of lithogenic material at each locations.

1.10 Column “References for PAl” gives the corresponding reference from which the Pal was taken for each location

1.11 Column “References for export fluxes” gives the corresponding reference from which the export fluxes were taken for each location
