# Supplementary material for: Where is mineral ballast important for surface export of particulate organic carbon in the ocean?
Source: Geophys Res Lett. 2014 Dec 3;41(23):8460–8. doi: 10.1002/2014GL061678 (PMC4459180; doi:10.1002/2014GL061678)
Supplement: Supplementary file 2 — Figures S1 and S2 and Table S1 [file grl0041-8460-sd2.docx]

Where is mineral ballast important for surface export of particulate organic carbon in the ocean?

Frédéric A.C. Le Moigne^1*^, Katsiaryna Pabortsava^2^, Charlotte L.J. Marcinko^1^, Patrick Martin^2,3^, Richard J. Sanders^1^.

^1^Ocean biogeochemistry and Ecosystems, National Oceanography Centre, Southampton, SO14 3ZH, UK; ^2^School of Ocean and Earth Science, University of Southampton, Southampton SO14 3ZH, UK. ^3^Earth Observatory of Singapore, Nanyang Technological University, 50 Nanyang Avenue, Singapore 639798.

**:* Corresponding author, Frédéric A.C. Le Moigne, [f.lemoigne@noc.ac.uk](mailto:f.lemoigne@noc.ac.uk); National Oceanography Centre, European Way, SO143ZH, Southampton, U.K.

**Supplementary information**

Table S1: Compilation of Th derived POC and mineral export fluxes data used in the regression analysis. NADR: North Atlantic drift province, SARC: Atlantic subarctic province, SANT: Subantartic province, SATL: South Atlantic gyral province, WTRA: Western tropical Atlantic province, NASE: North Atlantic subtropical gyral province east, ARCT: Atlantic arctic province, SSTC: South subtropical convergence province, NATR: North Atlantic tropical gyral province, APLR: Austral polar province, ANTA: Antarctic province.

| [[*Longhurst*, 1991](#_ENREF_1)] | Latitude | Longitude | POC export | PIC export | BSi export | Litho export | Surface temperature | Sampling date | PAl concentration | References for PAl | References for export fluxes |
| --- | --- | --- | --- | --- | --- | --- | --- | --- | --- | --- | --- |
| provinces |  |  | mg m^-2^ d^-1^ | mg m^-2^ d^-1^ | mg m^-2^ d^-1^ | mg m^-2^ d^-1^ | ºC | dd/mm/yyyy | nmol l^-1^ |  |  |
| NADR | 48.5 | -17.1 | 134.6 | 7.6 | 1.4 | 77.3 | 15.4 | 20/07/2009 | 3.9 | [*Kuss and Kremling*, 1999] | [*Le Moigne et al.,* 2013] |
| NADR | 49.1 | -16.6 | 207.2 | 11.6 | 2.2 | 119.0 | 15.4 | 23/07/2009 | 3.9 | [*Kuss and Kremling*, 1999] | [*Le Moigne et al.,* 2013] |
| NADR | 48.9 | -16.1 | 143.2 | 8.0 | 1.5 | 82.2 | 16.1 | 24/07/2009 | 3.9 | [*Kuss and Kremling*, 1999] | [*Le Moigne et al.,* 2013] |
| NADR | 49.0 | -16.9 | 101.9 | 13.2 | 1.1 | 78.2 | 15.1 | 29/07/2009 | 3.9 | [*Kuss and Kremling*, 1999] | [*Le Moigne et al.,* 2013] |
| NADR | 48.8 | -16.5 | 81.0 | 8.8 | 1.2 | 130.6 | 15.5 | 31/07/2009 | 3.9 | [*Kuss and Kremling*, 1999] | [*Le Moigne et al.,* 2013] |
| NADR | 49.0 | -16.4 | 50.5 | 10.0 | 0.3 | 45.8 | 15.3 | 03/08/2009 | 3.9 | [*Kuss and Kremling*, 1999] | [*Le Moigne et al.,* 2013] |
| NADR | 48.9 | -16.5 | 20.4 | 2.0 | 0.3 | 31.1 | 15.3 | 06/08/2009 | 3.9 | [*Kuss and Kremling*, 1999] | [*Le Moigne et al.,* 2013] |
| SARC | 59.7 | -20.5 | 225.4 | 66.5 | 84.3 | 207.3 | 13.5 | 29/07/2007 | 3.9 | [*Kuss and Kremling*, 1999] | [*Sanders et al.*, 2010] |
| SARC | 59.7 | -18.7 | 458.5 | 166.1 | 222.1 | 244.6 | 13.5 | 30/07/2007 | 3.9 | [*Kuss and Kremling*, 1999] | [*Sanders et al.*, 2010] |
| SARC | 58.9 | -21.0 | 294.3 | 82.2 | 92.5 | 111.9 | 13.4 | 02/08/2007 | 3.9 | [*Kuss and Kremling*, 1999] | [*Sanders et al.*, 2010] |
| SARC | 60.0 | -20.5 | 59.1 | 63.9 | 3.5 | 22.3 | 13.9 | 04/08/2007 | 3.9 | [*Kuss and Kremling*, 1999] | [*Sanders et al.*, 2010] |
| SARC | 59.2 | -19.1 | 53.5 | 44.3 | 56.4 | 27.4 | 14.0 | 09/08/2007 | 3.9 | [*Kuss and Kremling*, 1999] | [*Sanders et al.*, 2010] |
| SARC | 59.2 | -19.9 | 32.6 | 11.9 | 14.6 | 30.5 | 13.5 | 12/08/2007 | 3.9 | [*Kuss and Kremling*, 1999] | [*Sanders et al.*, 2010] |
| SARC | 59.7 | -18.7 | 194.3 | 52.3 | 46.7 | 164.8 | 13.0 | 14/08/2007 | 3.9 | [*Kuss and Kremling*, 1999] | [*Sanders et al.*, 2010] |
| SARC | 59.7 | -18.7 | 341.7 | 122.4 | 78.9 | 445.7 | 13.5 | 18/08/2007 | 3.9 | [*Kuss and Kremling*, 1999] | [*Sanders et al.*, 2010] |
| SARC | 59.3 | -19.8 | 159.7 | 43.0 | 38.4 | 135.6 | 12.9 | 19/08/2007 | 3.9 | [*Kuss and Kremling*, 1999] | [*Sanders et al.*, 2010] |
| SANT | -46.0 | 51.8 | 58.9 | 18.5 | 179.9 | 21.3 | 3.9 | 18/11/2004 | [*Planquette et al*., 2008] | [*Planquette et al*., 2008] | [*Morris et al.,* 2007] |
| SANT | -49.0 | 51.5 | 69.5 | 173.0 | 112.6 | 2.0 | 2.9 | 23/11/2004 | [*Planquette et al*., 2008] | [*Planquette et al*., 2008] | [*Morris et al.,* 2007] |
| SANT | -46.1 | 51.8 | 139.6 | 463.7 | 413.2 | 16.6 | 4.4 | 25/11/2004 | [*Planquette et al*., 2008] | [*Planquette et al*., 2008] | [*Morris et al.,* 2007] |
| SANT | -45.5 | 49.0 | 163.3 | 5.5 | 419.5 | 6.6 | 5.5 | 27/11/2004 | [*Planquette et al*., 2008] | [*Planquette et al*., 2008] | [*Morris et al.,* 2007] |
| SANT | -45.0 | 49.9 | 183.5 | 97.3 | 221.9 | 24.7 | 6.0 | 30/11/2004 | [*Planquette et al*., 2008] | [*Planquette et al*., 2008] | [*Morris et al.,* 2007] |
| SANT | -44.9 | 49.6 | 163.2 | 86.1 | 378.6 | 3.5 | 5.9 | 02/12/2004 | [*Planquette et al*., 2008] | [*Planquette et al*., 2008] | [*Morris et al.,* 2007] |
| SANT | -46.1 | 51.8 | 240.0 | 46.1 | 389.1 | 17.0 | 5.2 | 23/12/2004 | [*Planquette et al*., 2008] | [*Planquette et al*., 2008] | [*Morris et al.,* 2007] |
| SANT | -46.0 | 56.2 | 153.2 | 68.9 | 116.3 | 8.6 | 6.4 | 27/12/2004 | [*Planquette et al*., 2008] | [*Planquette et al*., 2008] | [*Morris et al.,* 2007] |
| SANT | -46.1 | 51.8 | 186.9 | 17.0 | 434.7 | 61.8 | 4.9 | 31/12/2004 | [*Planquette et al*., 2008] | [*Planquette et al*., 2008] | [*Morris et al.,* 2007] |
| SANT | -49.0 | 51.5 | 172.9 | 277.9 | 878.5 | 2.1 | 4.2 | 03/01/2005 | [*Planquette et al*., 2008] | [*Planquette et al*., 2008] | [*Morris et al.,* 2007] |
| SANT | -47.8 | 52.8 | 225.4 | 201.9 | 738.7 | 4.5 | 5.1 | 06/01/2005 | [*Planquette et al*., 2008] | [*Planquette et al*., 2008] | [*Morris et al.,* 2007] |
| SANT | -46.0 | 51.9 | 121.1 | 219.1 | 274.7 | 13.2 | 6.1 | 10/01/2005 | [*Planquette et al*., 2008] | [*Planquette et al*., 2008] | [*Morris et al.,* 2007] |
| SATL | -41.0 | -41.6 | 64.4 | 39.0 | 1.2 | 73.4 | 13.5 | 02/05/2004 | 2.6 | [*Lambert et al.*, 1984] | [*Thomalla et al.*, 2008] |
| SATL | -33.0 | -31.0 | 44.3 | 11.0 | 2.4 | 36.6 | 17.5 | 05/05/2004 | 2.6 | [*Lambert et al.*, 1984] | [*Thomalla et al.*, 2008] |
| SATL | -24.2 | -25.0 | 30.4 | 10.0 | 1.2 | 41.6 | 21.0 | 08/05/2004 | 2.6 | [*Lambert et al.*, 1984] | [*Thomalla et al.*, 2008] |
| SATL | -12.3 | -25.0 | 39.5 | 5.0 | 0.6 | 44.2 | 25.5 | 11/05/2004 | 2.6 | [*Lambert et al.*, 1984] | [*Thomalla et al.*, 2008] |
| WTRA | -0.9 | -25.0 | 76.0 | 34.0 | 0.6 | 246.3 | 28.0 | 14/05/2004 | 3.7 | [*Lambert et al.*, 1984] | [*Thomalla et al.*, 2008] |
| WTRA | 11.4 | -29.4 | 75.2 | 41.0 | 4.8 | 1264.3 | 28.0 | 17/05/2004 | 4.8 | [*Lambert et al.*, 1984] | [*Thomalla et al.*, 2008] |
| NASE | 38.7 | -16.4 | 141.2 | 61.1 | 14.4 | 1804.8 | 23.0 | 26/05/2004 | 4.8 | [*Lambert et al.*, 1984] | [*Thomalla et al.*, 2008] |
| NADR | 48.8 | -17.0 | 89.9 | 14.4 | 0.2 | 87.2 | 15.6 | 08/08/2009 | 3.9 | [*Kuss and Kremling*, 1999] | [*Thomalla et al.*, 2008] |
| ARCT | 59.6 | -28.6 | 362.7 | 98.8 | 208.1 | 322.2 | 8.4 | 04/05/2010 | 3.9 | [*Kuss and Kremling*, 1999] | [*Le Moigne et al.*, 2012] |
| ARCT | 59.6 | -26.1 | 247.5 | 174.3 | 114.3 | 296.1 | 8.7 | 05/05/2010 | 3.9 | [*Kuss and Kremling*, 1999] | [*Le Moigne et al.*, 2012] |
| SARC | 60.5 | -21.5 | 334.9 | 154.9 | 258.7 | 384.8 | 9.2 | 06/05/2010 | 3.9 | [*Kuss and Kremling*, 1999] | [*Le Moigne et al.*, 2012] |
| SARC | 61.5 | -21.0 | 144.6 | 8.3 | 214.7 | 139.1 | 12.7 | 13/07/2010 | 3.9 | [*Kuss and Kremling*, 1999] | [*Le Moigne et al.*, 2012] |
| SARC | 59.6 | -23.4 | 179.1 | 31.8 | 128.8 | 148.7 | 12.5 | 15/07/2010 | 3.9 | [*Kuss and Kremling*, 1999] | [*Le Moigne et al.*, 2012] |
| ARCT | 62.6 | -29.5 | 253.9 | 14.6 | 258.9 | 280.1 | 11.4 | 24/07/2010 | 3.9 | [*Kuss and Kremling*, 1999] | [*Le Moigne et al.*, 2012] |
| ARCT | 62.0 | -24.2 | 192.5 | 42.9 | 246.2 | 110.1 | 11.2 | 03/08/2010 | 3.9 | [*Kuss and Kremling*, 1999] | [*Le Moigne et al.*, 2012] |
| SARC | 61.1 | -20.5 | 373.5 | 23.3 | 185.3 | 113.7 | 11.4 | 04/08/2010 | 3.9 | [*Kuss and Kremling*, 1999] | [*Le Moigne et al.*, 2012] |
| SARC | 61.2 | -20.6 | 145.2 | 16.0 | 190.1 | 26.0 | 13.6 | 06/08/2010 | 3.9 | [*Kuss and Kremling*, 1999] | [*Le Moigne et al.*, 2012] |
| ARCT | 60.6 | -34.6 | 354.4 | 99.5 | 392.7 | 174.2 | 6.6 | 01/05/2010 | 3.9 | [*Kuss and Kremling*, 1999] | [*Le Moigne et al.*, 2012] |
| ARCT | 60.0 | -34.6 | 457.2 | 100.3 | 548.0 | 205.4 | 6.5 | 02/05/2010 | 3.9 | [*Kuss and Kremling*, 1999] | [*Le Moigne et al.*, 2012] |
| ARCT | 60.0 | -31.6 | 158.0 | 50.2 | 473.5 | 164.5 | 7.6 | 03/05/2010 | 3.9 | [*Kuss and Kremling*, 1999] | [*Le Moigne et al.*, 2012] |
| ARCT | 60.0 | -34.6 | 253.9 | 14.6 | 258.9 | 280.1 | 12.9 | 17/07/2010 | 3.9 | [*Kuss and Kremling*, 1999] | [*Le Moigne et al.*, 2012] |
| ARCT | 59.5 | -41.3 | 192.5 | 42.9 | 246.2 | 110.1 | 10.7 | 19/07/2010 | 3.9 | [*Kuss and Kremling*, 1999] | [*Le Moigne et al.*, 2012] |
| ARCT | 59.6 | -34.6 | 373.5 | 23.3 | 185.3 | 113.7 | 11.2 | 20/07/2010 | 3.9 | [*Kuss and Kremling*, 1999] | [*Le Moigne et al.*, 2012] |
| ARCT | 62.6 | -35.0 | 145.2 | 16.0 | 190.1 | 26.0 | 12.0 | 22/07/2010 | 3.9 | [*Kuss and Kremling*, 1999] | [*Le Moigne et al.*, 2012] |
| ARCT | 58.1 | -35.1 | 258.2 | 66.6 | 269.5 | 79.4 | 11.9 | 26/07/2010 | 3.9 | [*Kuss and Kremling*, 1999] | [*Le Moigne et al.*, 2012] |
| ARCT | 63.5 | -35.1 | 163.7 | 21.9 | 34.6 | 95.2 | 12.3 | 30/07/2010 | 3.9 | [*Kuss and Kremling*, 1999] | [*Le Moigne et al.*, 2012] |
| ARCT | 63.5 | -34.6 | 187.1 | 11.8 | 134.3 | 166.5 | 11.7 | 01/08/2010 | 3.9 | [*Kuss and Kremling*, 1999] | [*Le Moigne et al.*, 2012] |
| NATR | 12.6 | -17.7 | 102.1 | 9.1 | 28.7 | 108.7 | 19.9 | 22/02/2011 | 4.8 | [*Kuss and Kremling*, 1999] | [*Pabortsava et al.*, in prep] |
| NATR | 12.6 | -23.6 | 74.8 | 5.6 | 30.9 | 119.4 | 24.2 | 25/02/2011 | 4.8 | [*Kuss and Kremling*, 1999] | [*Pabortsava et al.*, in prep] |
| WTRA | -7.2 | -25.0 | 49.0 | 3.2 | 1.6 | 44.8 | 28.0 | 03/02/2011 | 4.8 | [*Kuss and Kremling*, 1999] | [*Pabortsava et al.*, in prep] |
| WTRA | 1.2 | -26.0 | 44.0 | 8.2 | 16.7 | 39.8 | 28.2 | 06/03/2011 | 4.8 | [*Kuss and Kremling*, 1999] | [*Pabortsava et al.*, in prep] |
| NATR | 8.3 | -28.3 | 63.3 | 3.6 | 46.0 | 6.4 | 25.8 | 10/03/2011 | 4.8 | [*Kuss and Kremling*, 1999] | [*Pabortsava et al.*, in prep] |
| NATR | 15.5 | -28.8 | 64.5 | 4.1 | 7.0 | 43.5 | 23.8 | 13/03/2011 | 4.8 | [*Kuss and Kremling*, 1999] | [*Pabortsava et al.*, in prep] |
| NATR | 19.2 | -28.1 | 30.7 | 3.1 | 4.7 | 49.0 | 23.0 | 15/03/2011 | 4.8 | [*Kuss and Kremling*, 1999] | [*Pabortsava et al.*, in prep] |
| SATL | -36.5 | 13.3 | 27.1 | 37.9 | 74.0 | 0.0 | 13.0 | 21/10/2010 | 4.2 | [*Lambert et al.*, 1984] | [*Martin*, unpublished] |
| SSTC | -40.0 | 0.9 | 21.1 | 18.0 | 10.2 | 0.0 | 10.7 | 28/10/2010 | 4.2 | [*Lambert et al.*, 1984] | [*Martin*, unpublished] |
| SSTC | -36.5 | 13.2 | 77.4 | 56.2 | 330.3 | 0.0 | 16.5 | 08/11/2010 | 4.2 | [*Lambert et al.*, 1984] | [*Martin*, unpublished] |
| SSTC | -39.3 | 7.7 | 167.0 | 99.8 | 110.4 | 0.0 | 13.8 | 15/11/2010 | 4.2 | [*Lambert et al.*, 1984] | [*Martin*, unpublished] |
| SATL | -36.3 | 13.1 | 27.7 | 31.5 | 71.3 | 37.1 | 20.3 | 29/12/2011 | 4.2 | [*Lambert et al.*, 1984] | [*Pabortsava,* unpublished] |
| SSTC | -40.0 | -16.5 | 47.2 | 6.8 | 15.9 | 70.9 | 16.4 | 10/01/2012 | 2.7 | [*Lambert et al.*, 1984] | [*Pabortsava,* unpublished] |
| SSTC | -40.0 | -42.4 | 141.0 | 12.6 | 24.3 | 126.9 | 18.4 | 18/01/2012 | 2.7 | [*Lambert et al.*, 1984] | [*Pabortsava,* unpublished] |
| SATL | -38.0 | -51.0 | 52.3 | 8.5 | 7.4 | 38.8 | 22.2 | 22/01/2012 | 2.7 | [*Lambert et al.*, 1984] | [*Pabortsava,* unpublished] |
| SARC | 60.1 | -6.4 | 142.7 | 63.0 | 3.1 | 71.9 | 10.4 | 05/06/2012 | 5.9 | [*Lambert et al.*, 1984] | [*Le Moigne et al.*, submitted] |
| ARCT | 74.1 | -4.1 | 74.6 | 7.9 | 5.6 | 8.4 | 1.0 | 12/05/2012 | 5.9 | [*Lambert et al.*, 1984] | [*Le Moigne et al.*, submitted] |
| ARCT | 76.1 | -2.3 | 41.2 | 8.3 | 17.5 | 3.0 | 1.7 | 13/05/2012 | 5.9 | [*Lambert et al.*, 1984] | [*Le Moigne et al.*, submitted] |
| ARCT | 78.1 | -5.6 | 21.9 | 1.0 | 1.0 | 9.5 | -1.5 | 16/05/2012 | 5.9 | [*Lambert et al.*, 1984] | [*Le Moigne et al.*, submitted] |
| ARCT | 77.5 | 1.2 | 106.4 | 5.7 | 5.5 | 5.3 | 2.5 | 19/05/2012 | 5.9 | [*Lambert et al.*, 1984] | [*Le Moigne et al.*, submitted] |
| SARC | 76.2 | 12.3 | 174.9 | 2.6 | 5.5 | 17.7 | 5.8 | 22/05/2012 | 5.9 | [*Lambert et al.*, 1984] | [*Le Moigne et al.*, submitted] |
| SARC | 71.8 | 17.9 | 67.9 | 4.7 | 6.5 | 8.9 | 7.9 | 25/05/2012 | 5.9 | [*Lambert et al.*, 1984] | [*Le Moigne et al.*, submitted] |
| ARCT | 71.4 | 8.3 | 69.0 | 77.9 | 1.4 | 12.3 | 6.8 | 26/05/2012 | 5.9 | [*Lambert et al.*, 1984] | [*Le Moigne et al.*, submitted] |
| ARCT | 71.4 | -1.2 | 82.0 | 7.4 | 4.8 | 8.4 | 5.9 | 27/05/2012 | 5.9 | [*Lambert et al.*, 1984] | [*Le Moigne et al.*, submitted] |
| ARCT | 71.4 | -10.4 | 77.1 | 21.5 | 12.5 | 11.3 | 3.3 | 28/05/2012 | 5.9 | [*Lambert et al.*, 1984] | [*Le Moigne et al.*, submitted] |
| ARCT | 68.4 | -10.3 | 153.5 | 38.3 | 5.6 | 11.8 | 4.3 | 29/05/2012 | 5.9 | [*Lambert et al.*, 1984] | [*Le Moigne et al.*, submitted] |
| ARCT | 67.5 | -16.3 | 209.3 | 3.9 | 10.6 | 20.1 | 7.0 | 30/05/2012 | 5.9 | [*Lambert et al.*, 1984] | [*Le Moigne et al.*, submitted] |
| ARCT | 67.2 | -24.0 | 226.4 | 75.0 | 21.7 | 9.2 | 4.6 | 01/06/2012 | 5.9 | [*Lambert et al.*, 1984] | [*Le Moigne et al.*, submitted] |
| APLR | -59.9 | -53.1 | 103.3 | 18.8 | 61.6 | 10.9 | 0.5 | 15/01/2013 | 0.23 | [*Planquette et al*., 2008] | [*Le Moigne et al.*, in prep] |
| APLR | -59.9 | -49.4 | 64.2 | 35.6 | 796.6 | 13.5 | -2.1 | 17/01/2013 | 0.23 | [*Planquette et al*., 2008] | [*Le Moigne et al.*, in prep] |
| APLR | -59.9 | -45.3 | 140.5 | 33.2 | 251.5 | 45.4 | -0.8 | 19/01/2013 | 0.23 | [*Planquette et al*., 2008] | [*Le Moigne et al.*, in prep] |
| ANTA | -57.8 | -42.8 | 238.9 | 14.6 | 268.4 | 5.0 | 1.2 | 20/01/2013 | 0.23 | [*Planquette et al*., 2008] | [*Le Moigne et al.*, in prep] |
| ANTA | -55.2 | -41.3 | 39.5 | 8.9 | 269.7 | 3.1 | 2.8 | 21/01/2013 | 0.23 | [*Planquette et al*., 2008] | [*Le Moigne et al.*, in prep] |
| ANTA | -52.7 | -40.5 | 257.5 | 4.3 | 685.9 | 6.3 | 3.1 | 22/01/2013 | 1.27 | [*Planquette et al*., 2008] | [*Le Moigne et al.*, in prep] |
| SANT | -51.2 | -37.5 | 377.2 | 15.7 | 108.0 | 22.7 | 4.5 | 24/01/2013 | 1.27 | [*Planquette et al*., 2008] | [*Le Moigne et al.*, in prep] |
| SANT | -51.6 | -34.7 | 187.2 | 20.1 | 722.4 | 30.4 | 3.3 | 28/01/2013 | 1.27 | [*Planquette et al*., 2008] | [*Le Moigne et al.*, in prep] |
| ANTA | -53.8 | -29.2 | 317.5 | 16.4 | 2037.2 | 40.7 | 1.3 | 29/01/2013 | 1.27 | [*Planquette et al*., 2008] | [*Le Moigne et al.*, in prep] |
| ANTA | -58.8 | -25.9 | 127.1 | 1.0 | 11.4 | 13.4 | 0.2 | 01/02/2013 | 1.27 | [*Planquette et al*., 2008] | [*Le Moigne et al.*, in prep] |
| ANTA | -63.5 | -25.3 | 101.7 | 8.7 | 379.8 | 4.5 | 0.0 | 03/02/2013 | 0.23 | [*Planquette et al*., 2008] | [*Le Moigne et al.*, in prep] |
| ANTA | -60.0 | -29.5 | 389.1 | 13.8 | 1046.0 | 3.3 | 0.2 | 05/02/2013 | 0.23 | [*Planquette et al*., 2008] | [*Le Moigne et al.*, in prep] |


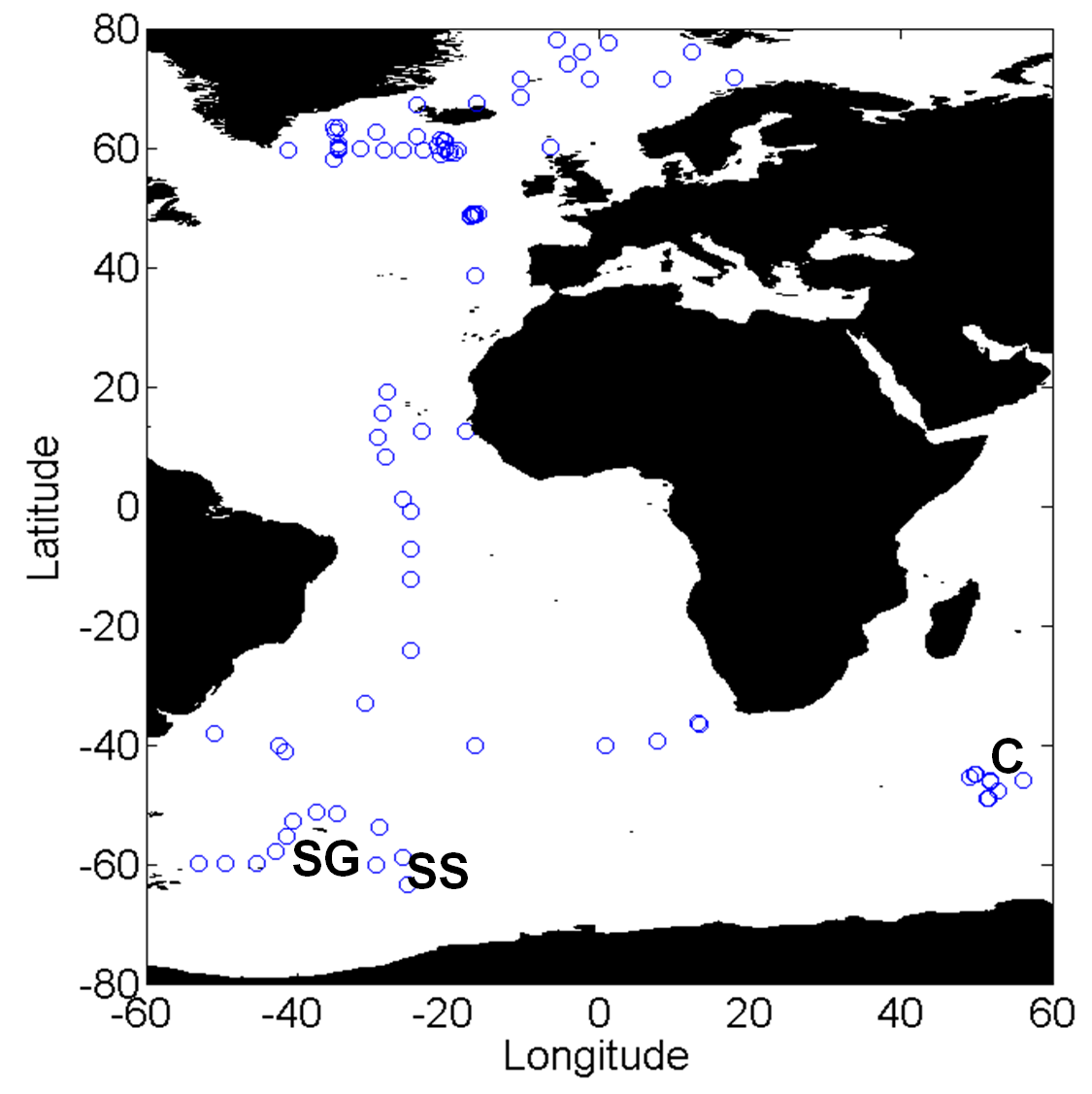


Figure S1: Sampling locations. Coordinates, sampling dates, fluxes data, corresponding [[*Longhurst*, 1991](#_ENREF_1)] provinces and references are given in Table S1. The locations of the Crozet (C), South Sandwich (SS) and South Georgia (SG) islands is indicated.


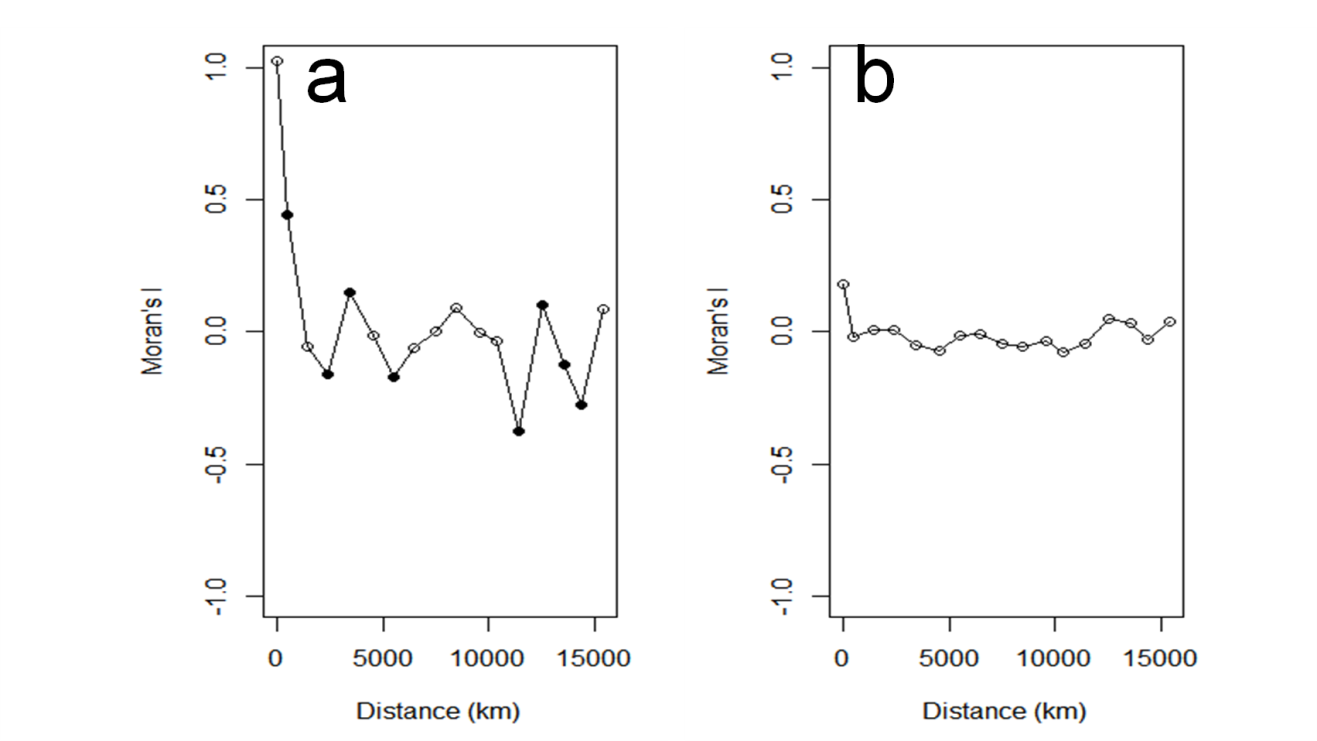


Figure S2: Correlograms comparing the spatial autocorrelation (Morans’s I) across distance for (a) MLRA and (b) GWR models. Significant residual spatial autocorrelation at a nominal (two-sided) 5%-level are shown as a filled circles, non-significant autocorrelation is shown as open circles. MLRA residuals show significant autocorrelation at several distance ranges indicating that response variables are influenced by the value of neighboring samples and that the assumption of independent observations is violated, leading to a potential bias in the confidence interval of Ccs obtained from this model, this is removed as a by-product of the GWR model.

References

Longhurst, A. R. (1991), Large marine ecosystems - Patterns, processes and yields., *Marine Policy*, *15*(5), 377-378.
